# Cardioprotection with sevoflurane during off-pump coronary artery surgery

**DOI:** 10.1186/1749-8090-8-S1-O158

**Published:** 2013-09-11

**Authors:** I Husedzinovic, N Bradic

**Affiliations:** 1 Clinic of Anesthesiology, Reanimatology and Intensive Medicine, University Hospital Dubrava, Zagreb, Croatia

## Objective

To evaluate a cardioprotective effect of sevoflurane in patients underwent off-pump coronary artery bypass grafting with normal preoperative left ventricular function.

## Methods

Study included 48 patients induced in anesthesia with 8 vol.% sevoflurane within high 100% oxygen flow (5 L/min). Patients randomized into two groups: sevoflurane group (n=24) in which anesthesia maintained with 1 MAC of end-tidal sevoflurane concentration, and propofol group (n=24), in which anesthesia maintained with continuous propofol infusion in doses of 2 to 3 mg/kg/h. Inclusion criteria were angiographically verified CAD and LVEF higher than 40%. Exclusion criteria were: AV conduction disturbances, AF with rapid ventricular response, myocardial infarction within 6 months, or diabetes mellitus. Pulmonary artery catheter used for the consecutive hemodynamic measurements. Cardiac index, heart rate, mean arterial pressure and central venous pressure were measured 5 minutes after anesthesia induction, on the beginning of ischemia, 15 minutes after ischemia and 15 minutes after sternum closure. For analysis between groups and time-points, a two-way analysis of variance (ANOVA) was performed and only p<0.05 was considered statistically significant.

## Results

There were no differences within group and between groups for the heart rate, mean arterial pressure and central venous pressure during surgery. There were no significant differences in cardiac index values within sevoflurane group. In propofol group significant decrease of cardiac index was on the beginning of ischemia (p<0.001). There were between-group differences in cardiac index values on the beginning of ischemia and 15 minutes after ischemia (p=0.002, and p=0.011, respectively).

## Conclusion

Cardiac function was preserved in patients anesthetized with sevoflurane comparing with patients treated with propofol.

